# ESLpred2: improved method for predicting subcellular localization of eukaryotic proteins

**DOI:** 10.1186/1471-2105-9-503

**Published:** 2008-11-28

**Authors:** Aarti Garg, Gajendra PS Raghava

**Affiliations:** 1 Department of Biotechnology, Panjab University, Chandigarh, India; 2 Bioinformatics Centre, Institute of Microbial Technology, Chandigarh, India

## Abstract

**Background:**

The expansion of raw protein sequence databases in the post genomic era and availability of fresh annotated sequences for major localizations particularly motivated us to introduce a new improved version of our previously forged eukaryotic subcellular localizations prediction method namely "ESLpred". Since, subcellular localization of a protein offers essential clues about its functioning, hence, availability of localization predictor would definitely aid and expedite the protein deciphering studies. However, robustness of a predictor is highly dependent on the superiority of dataset and extracted protein attributes; hence, it becomes imperative to improve the performance of presently available method using latest dataset and crucial input features.

**Results:**

Here, we describe augmentation in the prediction performance obtained for our most popular ESLpred method using new crucial features as an input to Support Vector Machine (SVM). In addition, recently available, highly non-redundant dataset encompassing three kingdoms specific protein sequence sets; 1198 fungi sequences, 2597 from animal and 491 plant sequences were also included in the present study. First, using the evolutionary information in the form of profile composition along with whole and N-terminal sequence composition as an input feature vector of 440 dimensions, overall accuracies of 72.7, 75.8 and 74.5% were achieved respectively after five-fold cross-validation. Further, enhancement in performance was observed when similarity search based results were coupled with whole and N-terminal sequence composition along with profile composition by yielding overall accuracies of 75.9, 80.8, 76.6% respectively; best accuracies reported till date on the same datasets.

**Conclusion:**

These results provide confidence about the reliability and accurate prediction of SVM modules generated in the present study using sequence and profile compositions along with similarity search based results. The presently developed modules are implemented as web server "ESLpred2" available at .

## Background

In this post genomic era, functional annotation and characterization of nearly millions of raw protein sequences, erupted by incredible sequencing projects, are some of the inescapable challenges that has been baffling the scientific community in order to bridge the mounting gap between number of unknown and annotated proteins. However, it is unfeasible to assign function to all proteins using purely time consuming and expensive experimental techniques, even if one compromises with experimental errors. Hence, this crisis entails the development of computational methods that would help in predicting functions of proteins expeditiously as well as economically. One of the fundamental and popular indirect strategies for assigning function is the identification of subcellular compartments of proteins as knowledge about localization can provide important indications about protein functions. After PSORT [1,2] the first method developed to predict the subcellular localizations, ample of novice, improved, generalized and organism specific prediction methods have been developed for predicting subcellular locations of eukaryotic and prokaryotic proteins, namely, NNPSL, PSORTB, FKNN, TargetP, SubLoc, SignalP, CELLO, LOCnet, PSLpred, HSLPred, PLOC, Mutiloc, Proteome Analyst, LOCtree, TSSub, BaCelLo and Esub8 using different datasets and protein input features [3-24]. In 2004, our group has combined the information of similarity search with sequence composition based attributes and achieved accuracy up to 88% [20]. Recently, it has been observed that use of multiple sequence alignment information in the form of Position Specific Scoring Matrix (PSSM) profiles predicted the subcellular localization of eukaryotic proteins with a higher accuracy [17,18,21]. Moreover, fusion of profile information (whole or N or C-terminals) with sequence compositional features resulted in the attainment of prediction accuracy upto 93% for eukaryotic subcellular localizations [17]. Hence, extraction of crucial protein attributes for the training of machine-learning technique is a key step to improve the prediction quality of protein subcellular localizations.

Indeed, PSSM based predictors have been able to achieve a very good performance but the dataset used to train some of these methods has been generated ~10 years back [3] and hence, not considered to be a very reliable for developing any new method. In particular, the growing sequence database and availability of newly annotated sequences for major localizations in the post genomic era, retrospectively encouraged us to introduce a new improved version of our previously forged eukaryotic subcellular localization prediction method ESLpred, trained on the same ~10 years older and highly redundant dataset (referred as RH2427 dataset [3]). Since, a need of predictors is only desired when no information can be inferred from homology-based search. Hence, the amount of redundancy present in the dataset used for developing subcellular localization prediction method is another issue, which has raised the questions on the robustness of the subcellular localization predictors, henceforth, demanding an evaluation on the highly non-redundant datasets. Therefore, an attempt has been made to include a recently generated highly non-redundant dataset (used for developing BaCelLo method [18]) as a major training set in the development of new "ESLpred2" method.

In a nut-shell, a systematic approach has been taken to improve the prediction quality of eukaryotic subcellular localizations using PSI-BLAST generated PSSM profiles along with compositional attributes and similarity search based information. These features are observed to be promising and highly crucial in predicting the localizations irrespective of the presence and absence of redundancy in the trained dataset. The present method has achieved a highest success rate for the prediction of localizations with good overall and average accuracy, and hence, compliments the existing subcellular localization prediction methods.

## Results and discussion

### Importance of evolutionary searching

Recently, the issues provoked by few reports regarding the usage of BLAST search information in prediction process have challenged the prediction accuracy of subcellular localizations methods [18,25], hence, necessitates evaluation of predictors without incorporating homology information. But the present authors feel that homology based searching is the first step for the functional annotation of unknown proteins. Further, this is considered to be the most reliable and promising method to elucidate the functions. Unfortunately, this technique fails when query proteins fail to find out significant homology within database. In that case, it is always profitable to add machine-learning based prediction results along with similarity based search information. Hence, we can say that machine learning based methods, indeed helping the homology based searching to provide more accurate predictions. But showing subcellular localization prediction accuracy just with homology based searching should not be recommendable.

### Need of a new improved version

ESLpred method, trained on RH2427 dataset [3] has been predicting the four types of eukaryotic sub localizations-cytoplasm, mitochondria, nuclear, and extracellular with a good overall accuracy of 88% since 2004. In addition, ESLpred has achieved highest success rate on the same dataset when compared with other popular methods such as SubLoc, NNPSL, Markov models and Fuzzy-k-NN. Though, LOCSVMPSI attained higher accuracy of 90%, nevertheless prediction accuracy of ESLpred for nuclear proteins observed to be much better. Although ESLpred has revealed hitherto success in prediction accuracy, the growing sequence databases and the requirement of adding new input feature which could have the potential to enhance the subcellular localizations prediction performance, necessitates the introduction of new version of ESLpred using recently developed highly non-redundant, kingdom specific datasets-animal with 2597 sequences; 1198 fungi sequences and 491 sequences were from plant [18] (referred as BaC2597, BaC1198, and BaC491 in subsequent discussions).

### Performance on RH2427 dataset

It is indispensable to show the prediction accuracy on RH2427 dataset which covered 4 major localizations such as cytoplasm, mitochondrial, nuclear and extracellular using new input features which were not implemented in ESLpred. The earlier method employed the hybrid approach based strategy of coupling amino acid composition, dipeptide composition, physicochemical properties and similarity search based information as an input features for the prediction of subcellular localizations.

Since, N-terminal signal is imperative for localizing certain classes of proteins mainly chloroplast, mitochondrial and extracellular to their final destinations, hence, the sequence of N-terminal can be used as a discriminating feature for the classification of proteins of these three classes from the rest. First, AAC-NTerm based SVM module was constructed, where the localizing signals present at N-terminal of the sequences were exclusively captured by calculating the amino acid composition of N-terminal along with whole sequence composition. The training of SVM model with this input feature of 40 dimensions, resulted an overall and average accuracy of 86.5% and 85.5% (kernel = RBF, γ = 20, C = 6) respectively as shown in Table 1.

**Table 1:** Detailed performance of various SVM based modules and EuPSI-BLAST on the RH2427 dataset

**Approaches**	**Cytoplasm**		**Mitochondria**		**Nuclear**		**Extracellular**		**Overall**		**Average**	
	ACC	MCC	ACC	MCC	ACC	MCC	ACC	MCC	ACC	MCC	ACC	MCC

AAC-NTerm (A)	82.2	0.76	83.5	0.82	90.3	0.80	86.2	0.86	86.5	0.80	85.5	0.81
PSSM based (B)	84.1	0.79	71.3	0.74	95.2	0.85	93.2	0.95	88.6	0.83	86.0	0.83
*EuPSI-BLAST (C)	77.6	---	54.8	---	84.5	---	86.7	---	---	---	---	---
Hybrid1 (A+B)	86.1	0.84	89.4	0.89	95.2	0.87	93.9	0.94	91.7	0.88	91.1	0.89
**Hybrid2 (A+B+C)**	**89.6**	**0.87**	**90.7**	**0.91**	**96.4**	**0.91**	**95.7**	**0.96**	**93.6**	**0.90**	**93.1**	**0.91**

ACC is accuracy; MCC is Matthew correlation coefficient

ACC is calculated in percentage

*The results are obtained from ESLpred method ^20^

Notably, the use of evolutionary information in the form of multiple sequence alignment profiles provides more information than a single sequence [26-34]. In addition, the subcellular localization methods based on PSSM also showed better performance than composition based methods [17,18,21]. Hence, in the present study, PSSM profiles were also used for the training of SVM models. It was observed that using 400 dimensional input vector of PSSM as such i.e. without normalization completely failed to classify test proteins into their respective classes. This failure might be due to presence of highly divergent scoring values present in the matrix. So it became apparent to scale down each matrix element in the range of 0–1 to have better classification. It was observed normalized PSSM based SVM module was able to accomplish good overall and average accuracy of 88.6 and 86% (γ = 30, C = 4) respectively as shown in Table 1, which is comparable to hybrid approach based SVM module of ESLpred. Furthermore, module was able to enhance the performance of subcellular localizations by 2% in comparison to accuracy achieved by presently developed sequence composition based modules. Hence, it shows that frequency of occurrence of each of the 20 amino acids at one position in the alignment is more informative than capturing the information from a single sequence. Importantly, it provides evidence about the reliability of PSSM profile as an input feature which alone could encapsulate the crucial attributes such as amino acid composition, local order, and evolutionary information for the prediction of subcellular localizations.

Further, with desire to enhance the performance, an attempt was made to combine the information of AAC-NTerm module with whole profile composition, which generated an input vector of 440 dimensions for the training of SVM model (referred as "hybid1 module"). The hybrid1 module astoundingly improved the prediction performance, exhibiting overall and average accuracy of 91.7 and 91.1% (γ = 10, C = 5) (Table 1), which is ~3% and 5% enhancement in prediction accuracies in comparison to PSSM based SVM module respectively.

Finally, coupling the similarity search based information (EuPSI-BLAST) with PSSM and AAC-NTerm based SVM module (hybrid2 module) enhanced the performance from 91.7% to 93.6% in terms of overall accuracy (Table 1). Moreover, an average accuracy for this module was 93.1%, which is best average accuracy every reported for RH2427 dataset. The accuracies achieved for cytoplasmic, mitochondrial, nuclear and extracellular classes were 89.6%, 90.7%, 96.4% and 95.7% respectively (Table 1). These results confirmed that our approach of using information, exploiting PSI-BLAST which generated both similarity based search results and PSSM profiles are crucial for detecting subcellular localization of proteins. Hence, usage of evolutionary information of proteins improved the prediction accuracy significantly in comparison to compositions based SVM modules.

### Comparison with ESLpred and other existing predictors for RH2427 dataset

ESLpred2 (trained on RH2427 dataset) is an improved version of our previous eukaryotic subcellular localization prediction method ESLpred. ESLpred method achieved an overall accuracy of 78.1%, 77.8%, 82.9% and 88% for modules based on amino acid composition, physico-chemical properties, dipeptide composition and the hybrid approach respectively. Further, nuclear, cytoplasmic, mitochondrial and extracellular proteins were able to be predicted with accuracies of 95.3%, 85.2%, 68.2% and 88.9% respectively using hybrid approach based module. In the present study, using the new input features (which were not implemented in ESLpred), ESLpred2 was able to attain better accuracy of ~94%, ~6% higher than that achieved by ESLpred, when trained on RH2427 dataset. The main credit for this achievement goes to the use of evolutionary information, amino acid composition along with similarity search based results as an input features. Moreover, increase in prediction accuracy was observed for all four major localizations also.

The comparison of the present method with recently developed subcellular localization methods also revealed good enhancement. First, we compared ESLpred2 (trained on RH2427 dataset) with LOCSVMPSI method, which was trained on the same dataset using PSSM along with amino acid composition as an input feature. LOCSVMPSI has achieved an overall accuracy of 90.2% with jackknife test. The results showed that overall prediction accuracy of ESLpred2 (using 5-fold cross-validation) was 4% higher, with better accuracy for each location also. For TSSub method, which integrated four different probabilistic neural network classifiers for four different features along with SVM classifier, overall prediction accuracy of ESLpred2 was ~1% higher and average accuracy was ~2% higher. For class-wise comparison, ESLpred2 attained ~2 and 6% higher accuracies for extracellular and mitochondrial classes. In a nut shell, overall prediction accuracy of ESLpred2 is 21%, 18%, 15%, 9%, 6%, 4% and 1% higher when compared with Markov model, NNPSL, SubLoc, Fuzzy k-NN, ESLpred, LOCSVMPSI, and TSSub methods respectively.

### Training on latest and highly non-redundant datasets

In addition, the present study was also carried out on highly non-redundant, three additional datasets, incorporated in order to have an idea about the effectiveness of the use of evolutionary information along with compositional features for discriminating subcellular localizations while using highly non-redundant dataset. For this notion to fulfill, we employed BaCelLo dataset, divided into three subsets to deduce the prediction accuracy on three kingdoms such as fungi, animals and plant proteins separately.

### Performance on BaC1198 dataset

Keeping in view, the importance of homology in functional annotation, similarity based search was carried out to encapsulate the evolutionary information of the proteins covering four major localizations such as cytoplasm, mitochondrial, nuclear and extracellular proteins. The module was able to yield an overall and average accuracy of 29.5% and 23.1% respectively, however, 780 proteins were not able produce any significant homology with the local database of fungi proteins after five-fold cross-validation. Therefore, in order to cover 100% predictions for 1198 proteins, it was indispensable to learn machine learning technique, or to fuse both.

Hence, we trained a SVM model with input features such as PSSM profile along with AAC-NTerm compositions (hybrid1), which eventually accomplished an overall accuracy of 72.7% along with higher average accuracy of 75.7% (kernel = RBF, γ = 5, C = 4) (Table 2). The comparison of hybrid1 module's performance with BaCelLo method revealed an improvement of ~3% in overall accuracy, whereas average accuracy was comparable as shown in Table 2. Hence, the present strategy of using evolutionary information in the form of PSSM profiles along with sequence compositional attributes seems to be a promising and crucial feature in increasing the prediction accuracy of subcellular localizations. Importantly, the strategy also works very well irrespective of the presence and absence of redundancy in the dataset.

**Table 2:** The detailed prediction results of different modules and comparison of performance with BaCelLo method on non-redundant and organism specific datasets

Datasets	Localizations	PSI-BLAST (A)		(PSSM+AAC-NTerm) (B)		Hybrid2 (A+B)		Hybrid2 (10-fold CV)		^#^Using BaCelLo strategy (B)	*BaCelLo^18 ^method*
		**ACC**	**MCC**	**ACC**	**MCC**	**ACC**	**MCC**	**ACC**	**MCC**	**ACC**	***ACC (Third level)***

**Fungi dataset**	*Cytoplasm*	10.9	----	53.6	0.32	54.0	0.36	51.7	0.37	62.6	*60.2*
	*Mitochondria*	12.2	----	84.0	0.75	82.5	0.77	83.5	0.77	90.4	*81.4*
	*Nuclear*	39.7	----	73.0	0.73	78.6	0.59	80.7	0.60	74.7	*67.1*
	*Extracellular*	29.6	----	92.1	0.92	92.1	0.93	93.2	0.93	94.3	*94.3*
	***Overall***	**29.5**	----	*72.7	**0.56**	**75.9**	**0.60**	**77.0**	**0.61**	**80.5**	*70.1*
	***Average***	**23.1**	----	*75.7	**0.63**	**76.8**	**0.66**	**77.3**	**0.67**	**76.5**	*75.8*

		**ACC**	**MCC**	**ACC**	**MCC**	**ACC**	**MCC**	**ACC**	**MCC**	**ACC**	***ACC (Third level)***

**Animal dataset**	*Cytoplasm*	28.7	*----*	62.9	0.42	63.3	0.49	61.3	0.48	70.6	*65.3*
	*Mitochondria*	17.0	----	77.1	0.75	78.2	0.77	78.7	0.77	91.5	*76.1*
	*Nuclear*	53.8	----	69.0	0.60	77.7	0.68	79.1	0.69	72.6	*64.8*
	*Extracellular*	40.9	----	92.4	0.86	95.3	0.90	95.0	0.90	93.8	*90.8*
	***Overall***	**42.9**	----	*75.8	**0.66**	**80.8**	**0.72**	**81.0**	**0.73**	**80.1**	*73.8*
	***Average***	**35.0**	----	*75.4	**0.66**	**78.6**	**0.71**	**78.5**	**0.71**	**82.1**	*74.2*

		**ACC**	**MCC**	**ACC**	**MCC**	**ACC**	**MCC**	**ACC**	**MCC**	**ACC**	***ACC (Fourth level)***

**Plant dataset**	*Chloroplast*	31.4	----	77.5	0.67	81.9	0.69	82.8	0.71	90.7	*73.0*
	*Cytoplasm*	6.90	----	51.7	0.50	50.0	0.53	50.0	0.50	79.3	*51.7*
	*Mitochondria*	16.4	----	67.2	0.66	65.8	0.63	70.2	0.66	67.2	*50.7*
	*Nuclear*	48.8	----	80.2	0.77	81.8	0.76	81.8	0.79	86.8	*71.9*
	*Extracellular*	26.8	----	87.8	0.65	90.2	0.70	95.1	0.76	85.4	*85.4*
	***Overall***	**30.3**	----	*74.5	**0.66**	**76.6**	**0.68**	**78.0**	**0.70**	**84.7**	*68.2*
	***Average***	**26.4**	----	*72.9	**0.64**	**73.9**	**0.67**	**76.0**	**0.69**	**81.9**	*66.6*

ACC is accuracy; MCC is Matthew correlation coefficient; ACC is calculated in percentage

*Overall and average accuracy obtained at SVM parameters: For Fungi dataset (kernel = RBF, γ = 5, C = 4); Animal dataset (kernel = RBF, γ = 5, C = 2); Plant dataset (RBF, γ = 9, C = 3).

^# ^SVM parameters obtained for each class using hybrid1 features-For Fungi dataset (Cytoplasm: j = 4, γ = 7, C = 0.4, threshold value = 0.0; Mitochondria: j = 5, γ = 1, C = 1.6, threshold value = 0.0; Nuclear: j = 4, γ = 7, C = 0.54, threshold value = 0.0; Extracellular: j = 3, γ = 1, C = 1, threshold value = 0.0), Animal dataset (Cytoplasm: j = 3, γ = 9, C = 0.5, threshold value = 0.0; Mitochondria: j = 25, γ = 1, C = 2, threshold value = 0.0; Nuclear: j = 3, γ = 9, C = 0.5, threshold value = 0.0; Extracellular: j = 6, γ = 2, C = 1, threshold value = -0.1), Plant dataset (Cytoplasm: j = 2, γ = 3, C = 0.7, threshold value = 0.1; Mitochondria: j = 1, γ = 5, C = 75, threshold value = 0.2; Nuclear: j = 2, γ = 3, C = 0.7, threshold value = 0.1; Extracellular: j = 9, γ = 1, C = 1, threshold value = 0.0)

Finally, addition of similarity search based information to hybrid of PSSM and AAC-NTerm resulted in further improvement of overall accuracy from 72.7% to 75.9%, and average accuracy from 75.7% to 76.8%, which is ~6% and ~1% better performance in comparison to BaCelLo method as shown in Table 2. Indeed, achieving better overall and average accuracies along with ~2% and ~12% astounding improvement in case of mitochondrial and nuclear protein in comparison to BaCelLo method, the prediction accuracy of cytoplasmic class was noticed to be very lower (~6%) as compared with the cytoplasmic class of BaCelLo method. The comprehensive analysis of accuracies for each class demonstrated a good coverage of 92.1%, 82.5% and 78.6% for extracellular, mitochondrial and nuclear classes respectively; however, cytoplasmic class found to be least discriminated with 54% of accuracy with our strategy of using input features. The possible reason for this poor performance of cytoplasmic class might be the use of composition of N-terminal signal along with whole sequence and profile composition that eventually resulted in overestimation of localization signal pertaining classes and masked the performance of signal lacking cytoplasmic class. But this observation was not realized while developing the same SVM based module for other dataset such as RH2427, where, cytoplasmic proteins were predicted with good accuracies along with N-terminal signal containing classes.

Therefore, to have broad insight, we also adopted the BaCelLo's architecture of building SVM layers arranged in a decision tree manner for prediction of classes. Here, an input features of 440 dimensions i.e. PSSM with AAC-NTerm compositions (hybrid1) was used. Doing so, extracellular, mitochondrial, cytoplasmic and nuclear proteins were predicted with 94.3%, 90.4%, 62.6%, and 74.7% of accuracies respectively at the third level of the decision tree (Table 2). Moreover, at the same level of the decision tree, we were able to attain overall and average accuracy of 80.5% and 76.5%, which is ~11% and 1% better performance in comparison to 70.1% and 75.8% achieved by BaCelLo method respectively.

This proves that use of 440 dimensional input features of PSSM along with whole and N-terminal sequence composition provided better discriminating results in terms of overall and average accuracies, whether, adopting our present strategy of constructing 1-v-r SVM models or BaCelLo's architecture of building SVM layers. Although cytoplasmic class achieved better performance when BaCelLo's strategy was adopted, which might be due to the fact that use of SVM layers in a decision tree manner, the complications and competitions between proteins to go and predict in different classes reduced a lot at each level. For instance, at the third level of BaCelLo's architecture, only two classes were left to be discriminated such as nuclear and cytoplasmic proteins, whereas, in our case (1-v-r strategy), all the four classes were simultaneously considered. Hence, an increase in accuracy for cytoplasmic class of fungi proteins was observed while using BaCelLo's architecture.

### Performance on BaC2597 dataset

This dataset also covered four major localizations such as cytoplasm, nuclear, mitochondrial and extracellular classes; however, protein sequences were exclusively from animal kingdom. First similarity search based module was developed using the dataset of 2597 proteins and the module was able to predict 1114 correct hits yielding an overall accuracy and average accuracy of 42.9 and 35% respectively. Next, hybrid1 module using whole profile compositions along with AAC-NTerm module was able to achieve better overall and average accuracy of 75.8% and 75.4% (kernel = RBF, γ = 5, C = 2), which is ~2 and 1% better performance than 73.8% and 74.2% yielded by BaCelLo method respectively (Table 2). However, on adding the similarity search based results to the hybrid1 module, astonishingly increase in accuracies was accomplished. The hybrid2 module achieved ~5% and ~4% better performance in comparison to hybrid1 module by achieving overall and average accuracies of 80.8% and 78.6%, respectively (Table 2). Further, comparison with BaCelLo method also showed better performance by our method with an increase of ~7% and 5% in overall and average accuracies respectively. Herein, the use of hybrid1 features for the building of SVM models in three layers as in BaCelLo's architecture, overall and average accuracies of 80.1% and 82.1% was achieved as shown in Table 2.

### Performance on BaC491 dataset

Unlikely BaC2597 and BaC11198 datasets, BaC491 dataset covered five major localizations such as chloroplast, cytoplasm, extracellular, mitochondrial and nuclear. In the present study, the similarity search based module also provided a poor coverage for plant proteins with achieving an overall and average accuracy of 30.3% and 26.4% respectively. Herein, the use of profile composition with AAC-NTerm module as an input feature for training of SVM module yielded 77.5%, 51.7%, 87.8%, 67.2% and 80.2% of accuracies for five classes respectively (Table 2). The module was able to attain overall and average accuracies of 74.5 and 72.9% (kernel = RBF, γ = 9, C = 3) respectively. Further, making the hybrid2 module using PSSM and AAC-NTerm based module along with similarity search based results, an increase in overall and average accuracies to 76.6% and 73.9% was observed. The comparison of performance with fourth level accuracies of BaCelLo's method revealed an enhancement of ~9% and 7% in overall and average accuracies respectively by our method. In addition, the present input features of 440 dimensions when used for constructing SVM models arranged four layers same as in BaCelLo's method, overall and average accuracies of 84.7 and 81.8% was attained respectively, which was observed to be an astounding increase of 17% and 15% of accuracies in comparison to BaCelLo's performance. Hence, our strategy of using evolutionary information in the form of 400 dimensional input vector, along with whole and N-terminal sequence compositions and similarity search based results seems to promising and reliable strategy of predicting subcellular localization. Importantly, the technique is able to predict localizations with higher accuracy even if data is highly non-redundant.

In addition, we also evaluated the performance of hybrid2 SVM model using the 10-fold CV evaluation technique and the accuracies were found to be slightly better in comparison to the accuracies obtained using 5-fold CV for plant and fungi protein dataset, whereas, for animal dataset the performance was observed to be nearly similar (Table 2). However, here, the direct comparison of these results with BaCelLo method cannot be made as different evaluation 10-fold CV procedure was adopted to assess the performance of SVM models.

Further, to assess the usage of validation set in SVM training as used earlier in the BaCelLo study, the dataset was divided into 5 sets-three sets were used for training, one as validation set (for selecting SVM parameters and optimal threshold value) and last one was used as testing set for 5-fold CV. Here, we also adopted BaCelLo's architecture of building SVM layers arranged in a decision tree manner. For animal dataset, it was found that overall and average accuracies of 79.8 and 81% was achieved, which was a slighter reduction from the earlier ones (80.1 and 82.1%) obtained without using the validation sets. This reduction might have arisen due to smaller size of training set left for training the SVM (3 out of 5) model in comparison to previous one where 4 out of 5 sets were used for training the model.

### Testing on independent datasets

The best way to judge the unbiased performance of any predictor is to assess its performance on an independent dataset. Here, we used the same independent dataset as used previously by Pierleoni et al [18] for comparing the performance of BaCelLo method with best publicly available subcellular localizations prediction methods. In order to have the fair comparison with existing methods, Pierleoni et al used the sequences upto Swiss-Prot version 41 for retraining the method and the remaining sequences till version 48 for independent testing on the retrained models. Thus, in the present study, to have reasonable comparison with other methods and BaCelLo itself, ESLpred2 was retrained with sequences upto Swiss-Prot version 41 and then tested on the same independent dataset of 707 and 179 animal and fungi protein sequences respectively. Since, independent dataset for plant proteins was very small, hence that was not included in the prediction process. The detailed evaluation of performances on these two datasets have already been shown by Pierleoni et al with best publicly available subcellular localizations prediction methods such as LOCtree, Psort II, SubLoc, ESLPred, and LOCSVMPSI and surprisingly better performance for BaCelLo methods was observed in comparison to other methods. Therefore, mainly the comparison of present method with BaCelLo method was performed in the present study.

For animal protein dataset, it was found that our method was able to predict 503 proteins out of 707 proteins correctly, yielding average and overall accuracies of 70.7 and 71.2% respectively as shown in Table 3. The comparison of ESLpred2 with BaCelLo method also revealed ~2% better accuracies along with enhanced performance for certain classes by our method. It was observed that ESLpred2 was able to achieve 2%, and 5%, better accuracies for nuclear and extracellular protein classes as compared to BaCelLo method (Table 3). Further, our method showed ~8%, 11% and 9% better overall accuracies when compared with LOCtree [15], PLOC [29] and MultiLoc [14] methods respectively. Moreover, PLOC method performed very badly on cytoplasmic, extracellular and mitochondrial proteins in comparison to ESLpred2 and BaCelLo methods. In this post genomic era, one can fully utilize the features of any prediction method only if it allows the prediction on whole genome/proteome rather than a single sequence at a time, this is the main reason that has limited the utility of some latest prediction methods such as ProLoc-GO, Cell-PLoc [31,32] which allow the prediction to be made on a single or few sequences only at a time. However, predictions on another most popular method Proteome Analyst [16] yielded an average accuracy of 83.2% on an independent dataset, which is significantly better than our present, LOCtree and BaCelLo methods. However, the present author feels this comparison to be unbiased as these predictions were completely based on the homology search of 707 proteins in the SWISSPROT database, and the presence of these proteins in the database likely to be predicted correctly by the method.

**Table 3:** The detailed evaluation of performance on an independent datasets of 707 animal and 179 fungi proteins

	Localizations	**ESLpred2**	BaCelLo*	LOCtree*	PLOC	MultiLoc
**Animal independent dataset**	Cytoplasm	**54.8**	54.0	38.2	23.4	60.6
	Mitochondria	**68.6**	68.6	60.0	54.2	65.7
	Nuclear	**68.0**	66.1	62.2	82.1	58.4
	Extracellular	**91.3**	85.5	84.9	42.4	68
	***Overall Accuracy***	**71.2**	68.6	63.0	59.7	61.5
	***Average Accuracy***	**70.7**	68.5	61.3	50.5	63.2

	Localizations	**ESLpred2**	BaCelLo*	LOCtree*	PLOC	MultiLoc

**Fungi independent dataset**	Cytoplasm	**26.7**	56.7	46.7	13.3	20.0
	Mitochondria	**90.9**	100	63.6	45.5	72.7
	Nuclear	**88.5**	66.4	66.4	87.7	54.9
	Extracellular	**100**	93.8	81.3	62.5	75.0
	***Overall Accuracy***	**79.3**	69.2	64.3	70.4	52.0
	***Average Accuracy***	**76.5**	79.2	64.3	52.3	55.7

*The values are obtained from reference 18

In the case of 179 fungi proteins, ESLpred2 was able to achieve overall and average accuracies of 79.3% and 76.5% respectively. Although the present method was able to attain 10% better overall accuracy in comparison to BaCelLo method, still, 2% lower performance in terms of average accuracy was observed for our method (Table 3). Again the present method was able to achieve much better accuracies in comparison to other eukaryotic subcellular localization prediction methods such as LOCtree, PLOC and MultiLoc, hence, increasing the confidence about the reliability and robustness of present method in comparison to others.

### ESLpred2 server

The SVM modules constructed in the present study have been implemented as World Wide Web server "ESLpred2", available at  using CGI/Perl script. The server runs on SUN server 420R under the Solaris environment. It is user-friendly web server and allows users to enter multiple protein sequence in the fasta format. Users can input protein sequences by pasting in the box or by using the file upload facility. The server also provides the options to select different approaches such as amino composition based, PSSM based and hybrid approach based SVM modules. In addition, there is a provision of selecting models trained on different datasets such as RH2427 and organism specific datasets (animal, plant and fungi). The prediction results consist of classification of the respective input sequences into its predicted subcellular localization along with SVM predicted scores.

## Conclusion

To conclude, an improved version of prediction of eukaryotic subcellular localization is presented here, covering four major localizations, coupled with kingdom specific prediction SVM models. An interesting feature of the present method is the hybrid of different protein features, such as composition of PSSM profile, whole and N-terminal composition of sequence and similarity search based results, which supported the assignment of the subcellular localization of proteins more reliably and with high accuracy irrespective of redundancy in the training datasets. The present method is able to complement all existing subcellular location prediction methods and provides an alternative way for biologists to predict protein subcellular locations.

## Methods

### Data set

The present method was trained using the latest dataset, which was earlier used for developing BaCelLo method [18]. The dataset was retrieved from SWISSPROT version 48.0 and divided into three subsets on the basis of kingdoms-animal with 2597 sequences; fungi with 1198 sequences and 491 sequences were from plant. The major attraction of this dataset was the stringent cut-off value of 30% used to reduce the similarity between sequences. The first two datasets covered 4 major localizations such as cytoplasm, mitochondria, nuclear, and extracellular, whereas, plant dataset included chloroplast class along with four major localizations. These three datasets has been referred as BaC2597, BaC1198, and BaC491 in our discussions.

### Support Vector Machine

In the present study, a freely downloadable package of SVM, SVM_light  was used to implement SVM. The prediction of subcellular localization is a multi-class classification problem, thus, 1 *vs *rest (1-v-r) approach was adopted, where, one class of proteins were labeled positive and proteins of remaining classes were labeled negative for the training of SVM model.

### Feature Vectors

#### Composition based features

Amino acid composition (AAC) is a fraction of 20 types of amino acids present in a protein sequence. It generates an input vector of 20 dimensions. Furthermore, to capture effectiveness of the signals present at N-terminals, amino acid composition of residues present at N-terminal (20 residues) along with whole sequence composition was calculated that eventually resulted an input vector of 40 dimensions (referred as AAC-NTerm).

#### Evolutionary information in the form of PSSM profiles

An attempt was made to use PSI-BLAST generated PSSM profile as an input feature for the training of SVM model [33]. For each sequence, PSI-BLAST search was carried out against non-redundant dataset available at SWISSPROT. After three iterations with cut-off E-value of 0.001, it generated a PSSM having the highest score as a part of the prediction process. The matrix consisted of 20 × M elements, where M is the length of the target sequence, and each element represents the frequency of occurrence of each of the 20 amino acids at one position in the alignment [29].

Further, in order, to make an input of fixed length, each element of the matrix (20 × M) was first scaled to the range of 0–1 using sigmoid function. Then, these normalized PSSMs (20 × M) were used to generate a 400-dimensional input vector by adding all the elements of rows in the PSSM corresponding to the same amino acids in the sequence. Finally, composition was calculated by dividing each element by the length of the protein sequence which provided a matrix of 20 × 20 elements.

#### Similarity search based module

Besides using PSI-BLAST to generate PSSM, it was also used to carry out similarity based search against the local in-built database of different localizations. For BaC2597, BaC1198 and BaC491 dataset, new modules were generated by carrying out similarity based search against the local datasets of 2597, 1198 and 491 proteins respectively. Three iterations of PSI-BLAST were carried out at a cut-off *E *value of 1 × 10^-5^. First, the dataset was divided into 5 sets, then four sets were used to build the database and the remaining fifth set was used for searching. This cycle was repeated five times so that each set can be used for searching the corresponding database of four sets. This module could predict any of the localizations, depending upon the similarity of the query protein to the proteins present in the database. The module would return "unknown subcellular localization" if no significant similarity was obtained.

#### Five-fold cross-validation

The performance of the modules constructed in this report was evaluated using 5-fold cross-validation technique. In this technique, the dataset was divided into 5 sets consisting of nearly equal number of sequences. These 5 sets were further portioned into training and test sets as shown in Figure S1 [see Additional file 1]. The training and testing was carried out five times at one particular values of C and γ, each time using one distinct set for testing and the remaining four sets for training. The final performance was obtained by averaging the performance of all five test sets.

The optimization of SVM model was carried out by selecting the well-known three types of kernel functions (such as linear, polynomial, RBF) with their corresponding optimization parameter selection search. For linear kernel, the search was carried out for the best value of C parameter; the values for parameters C and γ for RBF; and C and d for polynomial kernel to achieve high accuracy. While testing the value for γ parameter, regularization parameter C was kept at default value. Once the best parameters for both γ and C were found, then we did the fine tuning search surrounding the best values within the range of ± 5.

#### Evaluation parameters

To assess the predictive performance, evaluation parameters such as accuracy and Matthew correlation coefficient (MCC) were calculated using equation 1 and 2.

(1) $$\text{Accuracy (}x\text{)}=\frac{p\left(x\right)}{Exp\left(x\right)}$$

(2) $$\text{MCC }(x)=\frac{p\left(x\right)\;n\left(x\right)-u\left(x\right)\;o\left(x\right)}{\sqrt{\left[p\left(x\right)+u\left(x\right)\right]\;\left[p\left(x\right)+o\left(x\right)\right]\;\left[n\left(x\right)+u\left(x\right)\right]\;\left[n\left(x\right)+o\left(x\right)\right]}}$$

Where, *x *can be any subcellular location, Exp(*x*) is the number of sequences observed in location *x*, p(*x*) is the number of correctly predicted sequences of location *x*, n(*x*) is the number of correctly predicted sequences not of location *x*, u(*x*) is the number of under-predicted sequences and o(*x*) is the number of over-predicted sequences.

## Abbreviations

SVM: Support Vector Machine; PSSM: Position specific scoring matrix; AAC: Amino acid compositions; RBF: Radial basis function; MCC: Matthews Correlation Coefficient.

## Authors' contributions

AG developed different SVM modules, wrote computer programs, carried out data analysis and developed web server. GPSR conceived the project, coordinated it and refined the manuscript wrote by AG.

## Supplementary Material

Additional file 1 **The division of datasets into 5 sets to create training and test sets (Figure S1).** The figure provides an outline to divide the datasets into sub sets to carry out 5-fold CV using validation set. Click here for file
