# Supplementary figures and images for: ESLpred2: improved method for predicting subcellular localization of eukaryotic proteins
Source: BMC Bioinformatics. 2008 Nov 28;9:503. doi: 10.1186/1471-2105-9-503 (PMC2612013; doi:10.1186/1471-2105-9-503)

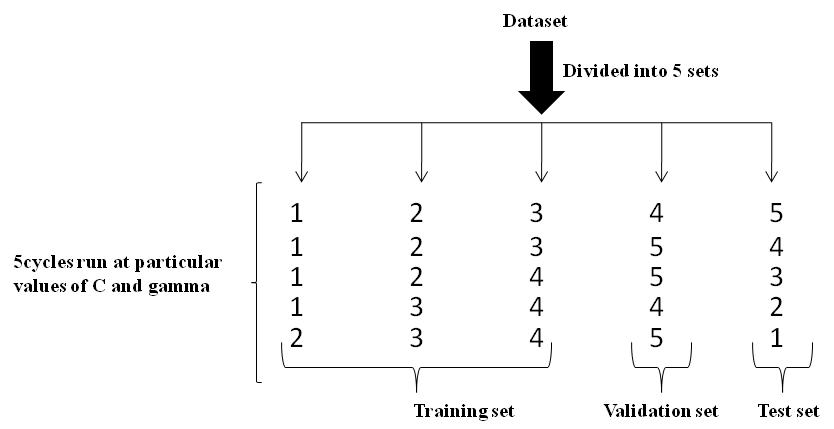
Figure S1. The division of datasets into 5 sets to create training and test sets

Supplement: Additional file 1 — The division of datasets into 5 sets to create training and test sets (Figure S1). The figure provides an outline to divide the datasets into sub sets to carry out 5-fold CV using validation set. [file 1471-2105-9-503-S1.doc]
